# A timed activity protocol to address sleep-wake disorders in home dwelling persons living with dementia: the healthy patterns clinical trial

**DOI:** 10.1186/s12877-021-02397-2

**Published:** 2021-08-03

**Authors:** Nancy A. Hodgson, Nalaka Gooneratne, Adriana Perez, Sonia Talwar, Liming Huang

**Affiliations:** 1 School of Nursing, University of Pennsylvania, Fagin Hall, Curie Blvd, Philadelphia, PA 19102 USA; 2 School of Medicine, University of Pennsylvania, Philadelphia, PA USA

**Keywords:** Dementia, Family caregiving, Sleep-wake disorders, Quality of life, Nonpharmacological strategies

## Abstract

**Background:**

Sleep-wake disorders occur in most persons living with dementia and include late afternoon or evening agitation, irregular sleep-wake rhythms such as daytime hypersomnia, frequent night awakenings, and poor sleep efficiency. Sleep-wake disorders pose a great burden to family caregivers, and are the principal causes of distress, poor quality of life, and institutionalization. Regulating the sleep-wake cycle through the use of light and activity has been shown to alter core clock processes and suggests that a combination of cognitive, physical, and sensory-based activities, delivered at strategic times, may be an effective mechanism through which to reduce sleep-wake disorders.

**Methods:**

A definitive Phase III efficacy trial of the Healthy Patterns intervention, a home-based activity intervention designed to improve sleep-wake disorders and quality of life, is being conducted using a randomized two-group parallel design of 200 people living with dementia and their caregivers (dyads). Specific components of this one-month, home-based intervention involve 4 in-home visits and includes: 1) assessing individuals’ functional status and interests; 2) educating caregivers on environmental cues to promote activity and sleep; and 3) training caregivers in using timed morning, afternoon, and evening activities based on circadian needs across the day. The patient focused outcomes of interest are quality of life, measures of sleep assessed by objective and subjective indicators including actigraphy, subjective sleep quality, and the presence of neuropsychiatric symptoms. Caregiver outcomes of interest are quality of life, burden, confidence using activities, and sleep disruption. Salivary measures of cortisol and melatonin are collected to assess potential intervention mechanisms.

**Discussion:**

The results from the ongoing study will provide fundamental new knowledge regarding the effects of timing activity participation based on diurnal needs and the mechanisms underlying timed interventions which can lead to a structured, replicable treatment protocol for use with this growing population of persons living with dementia**.**

**Clinical trial registration:**

Clinicaltrials.gov # NCT03682185 at https://clinicaltrials.gov/; Date of clinical trial registration: 24 September 2018.

## Background

Over 5 million Americans have Alzheimer’s disease or a related dementia, a progressive neurodegenerative condition, affecting over 15.5 million family caregivers [1, 2]. Sleep-wake disorders (SWD) occur in over 60% of persons living with dementia (PLWD) and are associated with increased morbidity, mortality, and poor quality of life [3]. Symptoms of SWD include late afternoon/evening agitation (e.g., “sundowning”), daytime hypersomnia, frequent night wakenings, and poor sleep efficiency [4]. SWDs place a tremendous burden on family caregivers, and are the leading causes of distress, poor quality of life (QOL), and institutionalization [4, 5] .

Disrupted sleep wake patterns in PLWD are thought to result from neurodegeneration in the suprachiasmatic nucleus neurons that drive the circadian rhythms of various physiological functions, such as the neuroendocrine (hypothalamic- pituitary-adrenal or HPA) and autonomic systems [6, 7]. In healthy adults HPA axis activity peaks between 0900 h–1100 h and coincides with high capacity and need for cognitively stimulating activity. HPA axis activity then follows a diurnal pattern that involves gradual decline throughout the day with a small rise between 1600 h–1900 h identified as an ideal time for physical activity [8]. The HPA axis activity nadir, or low point, typically occurs in the late evening prior to the onset of deep sleep and coincides with a peak in melatonin levels [9]. A flattening of HPA axis activity or a persistent rise in evening HPA activity in PLWD is associated with SWD [10]. Disruptions to sleep are further hastened in PLWD due to the diminished ability to respond to environmental cues (or zeitgebers) such as light (photic stimuli) and structured activity (nonphotic stimuli) that regulate or entrain the circadian clock [11, 12].

Given the potential harmful side effects of pharmacologic treatment for SWD in dementia, non-pharmacologic approaches may provide a safer alternative [13]. Regulating the sleep-wake system through the use of light and activity has been shown to alter core clock processes and suggests that specific activities delivered at strategic times may be an effective mechanism through which to entrain (or reset) disrupted sleep-wake rhythms for PLWD [14]. To date, most nonpharmacologic trials for SWD symptoms have focused on the administration of artificial indoor light because bright light is the strongest entraining agent for the circadian clock [15]. While studies of bright light therapy have demonstrated modest improvements in SWDs, they can be poorly tolerated by PWD [16] . A growing body of research support the importance of activity-based interventions as a nonpharmacologic approach to reduce the frequency and intensity of neuropsychiatric symptoms, enhance personhood, and improve QOL [17, 18] In particular, activities tailored specifically to a person’s own interests and functional level generate greater levels of engagement and positive outcomes [19].

A systematic review of 33 activity interventions showed improvement in QOL and reduction in neuropsychiatric symptoms [20]. Nevertheless, these studies almost exclusively involved non-experimental design, small sample sizes, nursing home residents, and did not include family caregivers or systematically measure sleep-wake patterns. Specific activity-based interventions intending to address SWDs have been conducted in the inpatient or nursing home setting where trained staff are available around the clock [21] and therefore may not be generalizable to the home setting- where most individuals live and prefer to remain. Existing research to elucidate the physiologic mechanism underlying the effect of activity-based interventions has been primarily focused on neurocognitive plasticity mechanisms rather than mechanisms explaining the effect of activity on neuropsychiatric symptoms [22]. Consequently, while activity for PWD is shown to be important in reducing neuropsychiatric behaviors and improving QOL, its optimal therapeutic use including the timing and structure of various types of activities remains unknown [23].

Systematic reviews of nonpharmacologic interventions to reduce sleep disruption have reported conflicting efficacy. One analysis found that sensory activity such as aromatherapy, warm baths, or gentle massage were efficacious as part of evening environmental interventions that promote relaxation and sleep [24]. A second review found that structured physical activity or social activity may help provide temporal cues to provide meaningful daytime stimulation and reduce daytime napping [25]. These reviews focused on sleep measures and emphasized the need for multi-modal approaches including evening sensory-environmental interventions that promote healthy sleep wake patterns. Importantly, most of the interventions in these reviews included only subjective measures of sleep and stress, as opposed to objective measures, or did not use a placebo control or other methodological rigors, thus making interpretation of study findings difficult.

To address this gap, the Healthy Patterns protocol was designed to test the efficacy of a timed and planned activity intervention designed to improve SWD and QOL, and to determine the underlying mechanisms by which the intervention achieves its potential benefits. Our primary hypothesis is that compared to attention control group participants, PLWD receiving the Healthy Patterns intervention will demonstrate improved quality of life, sleep efficiency, nocturnal wake after sleep onset, day/night sleep ratio, and neuropsychiatric symptoms at 1 month. Our secondary hypothesis is that compared to control group participants, caregivers who have received the “Healthy Patterns” training will demonstrate improved quality of life, and reduced burden, and sleep disruption at 1 month. Our third hypothesis explores the mediating effect of neuroendocrine activity (HPA axis) on changes in SWD. We hypothesis that SWDs will be mediated by changes in diurnal salivary cortisol and salivary melatonin from baseline to 1 month.

## Methods

### Study design

Reporting the design of this study follows CONSORT guidelines [26]. The study is a two-group, randomized, parallel design, clinical trial of 200 dyads of persons with dementia and their primary family caregiver. The study will evaluate the effectiveness of Healthy Patterns intervention compared with the attention control group and investigate the mechanisms by which SWDs are influenced by the timed activity intervention. Interested dementia caregivers who contact the research team will be screened for eligibility by telephone. Those dyads initially eligible will receive a nurse assessment to obtain written informed consent and to rule out primary sleep disorders requiring specialty care. Once eligibility is determined, dyads will receive a baseline home interview (T1). Following the baseline interview, dyads will be randomly assigned to experimental or attention-control group conditions. They will be contacted to be informed of their respective group assignment within 48 h of randomization. Experimental dyads will receive up to 4 in-home visits and 4 telephone contacts with a trained interventionist over 1-month. The attention-control group dyads will receive comparable attention in a structured program involving the same number and mix of in-home and telephone contact as the treatment group. All dyads will be retested at 1-month from baseline (T2) to evaluate proximal outcomes of SWDs, and QOL; and at 4 months to evaluate distal outcomes including QOL and other measures of satisfaction with the study procedures. Outcome measures will be assessed by interviewers masked to treatment assignment.

**Fig. 1:**
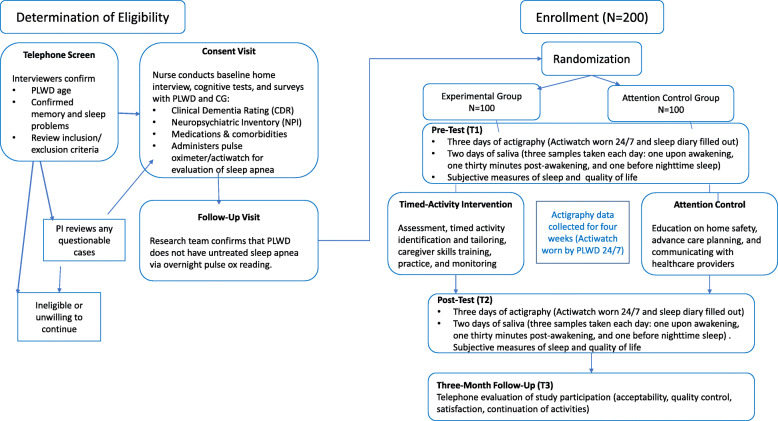
Study Flow

Figure 1 outlines the study flow. This study was approved by the University of Pennsylvania Institutional Review Board.

### Inclusion of participants

Our primary recruitment strategy involves identifying potential study participants from three possible sources: 1) A subject registry of individuals with dementia and their caregivers who participated in the study team’s previous studies, and have indicated a willingness to be contacted for future studies; 2) public advertising on public transit and local newspapers; 3) outreach to over 50 community-based programs and clinical services in the Philadelphia region serving cognitively frail individuals and with whom the study team has a recruitment partnership. Persons screened potentially eligible by telephone and willing to participate will be scheduled to receive a home assessment and verbal consent visit by a study nurse within a 10-day window.

#### Key inclusion criteria

Inclusion criteria for PLWD: (1) over age 60; (2) English speaking; (3) be able to tolerate wrist actigraphy and saliva collection procedures; (4) has caregiver reporting the presence of SWD symptoms [26]; and (5) diagnosed with probable dementia using standard assessments [27]. In addition, if the PLWD is on psychotropic medications (antidepressant, benzodiazepines, antipsychotic, or anti-convulsant) or an anti-dementia medication (memantine or a cholinesterase inhibitor), we will require that he/she will have been on a stable dose for 90 days prior to his/her enrollment to minimize possible confounding effects of concomitant medications and in line with typical time frames in dementia clinical trials.

#### Key exclusion criteria

Exclusion criteria for PLWD: (1) deemed to be in an unsafe situation at baseline; (2) planned transition to another residential care setting in 6 months or less; (3) at end-stage disease (defined as bed-bound and noncommunicative, or on hospice at baseline); (4) currently enrolled in another interventional clinical trial for dementia; (5) diagnosed with conditions known to affect measurement of circadian rhythm such as Huntington’s disease, Cushing’s disease, Addison’s disease, normal pressure hydrocephalus, Parkinson’s disease, advanced heart failure (New York Heart Stage 3–4), or morbid obesity (BMI > 35); (6)) current use of medications with substantial known effects on the measurement of HPA activity (e.g. corticosteroids, interferons, beta-blockers, cytotoxic chemotherapy); (7) presence of conditions with potential effects on HPA activity measurement e.g., major surgery in the past 3 months, major psychiatric disorder, history of heavy cigarette smoking (e.g. than 50 pack years), loss of a loved one in the past 3 months.

Telephone Screening Assent: First, dyads contacting the research team are screened to determine initial potential eligibility using a 10-min telephone screen. The presence of one or more symptoms of a sleep-wake disorder occurring in the PLWD several times a week over the past month will be used as the cut off for potential eligibility. These criteria will maximize the number of individuals who are eligible in the clinical home screening stage. Assent for telephone screen (based on an IRB approved telephone screening form and interviewer script) will be obtained from PLWD and caregiver during the initial phone screen by a trained study coordinator or research assistant. If PLWDs cognitive impairment prevents him/her from accurately recounting the purpose of the screen immediately following the description, the interviewer will ask to speak with the caregiver, to obtain proxy phone screening assent. The objectives, procedures, and a clear statement explaining risks and benefits of this study will be presented at the telephone screen. We will mail consent forms out immediately following a positive telephone screen, so that there is time to review the form prior to the in-person written consent procedures.

Written Consent: Dyads screened potentially eligible by telephone and willing to participate will be scheduled to receive a home assessment and consent visit. Written informed consent will be obtained for both the PLWD and their caregiver at the beginning of the in-home visit. The consent form, written in large print, will assure participants that their participation is voluntary. Every reasonable effort will be made to ensure that participants with sensory deficits have access to any and all medical devices and aides (e.g., reading glasses, hearing aids with functioning battery) during the consent procedure and during study visits. For those with language deficits, every reasonable attempt will be made ensure adequate communication. The consent designee will have the discretion to terminate the interview if he/she believes that communication is too impaired to ensure proper consent procedures. If the PLWD is too impaired to provide written consent, proxy written consent will be obtained from the legally authorized representative, and oral assent will be obtained from the PLWD. PLWD and their caregivers will receive an explicit assessment of capacity and the consent procedure will be documented by the study’s consent designee.

Following consent, a study nurse will then conduct a structured and standard clinical examination to: 1) obtain history of cognitive function and cognitive decline, pattern of losses, behavioral changes, and current functioning; 2) substantiate dementia diagnosis and disease stage using standardized mental, general physical, and neurological examinations including the Clinical Dementia Rating Scale (CDR-0.5 = very mild dementia; CDR-1 = mild; CDR-2 = moderate; CDR-3 = severe), 3) obtain medical history, co-morbidities, and medication use; and 4) establish presence of sleep disturbance [27].

#### Screen for sleep disordered breathing

Prior to randomization all screened and consented PLWD will have 1 night (approximately 9:00 p.m. to 6:00 a.m. on a night during no other research activities) of pulse oximetry to estimate the presence of sleep-disordered breathing, using a Nonin® WristOx2 Pulse Oximeter. This device is a wrist actigraph, outfitted with an integrated array of respiratory sensors, to reliably screen for sleep apnea. Given the potential challenges of the dementia population and in order to diminish obtrusiveness and enhance study feasibility, the configuration will only include the actigraph, and a fingertip oximeter (soft rubber sleeve). A nocturnal oxygen desaturation index (ODI) will be calculated as the number of oxygen desaturation events per hour of recording for each participant with at least 3 h of pulse oximetry. The Nonin® WristOx2 Pulse Oximeter has a sensitivity of 90.2%, and a specificity of 95.2% [28]. Using an ODI cutoff of 10 appears to optimize the sensitivity of identification of patients with sleep-disordered breathing versus patients without sleep-disordered breathing in sleep clinic samples. The treatment of sleep apnea is beyond the scope of the Healthy Patterns intervention and so participants with suspected sleep-disordered breathing will receive printed results of their ODI and instructions to seek a sleep study examination.

### Power calculation

#### Sample size

Sample size calculations are based on our ability to detect a medium effect size (d) of 0.45; and d) a type I error rate of .05. Clinical trials on symptoms of SWD use a medium effect size as an indication of clinical significance. To attain 80% power for a two-sided comparison of the two treatment groups at 1 month will require 78 dyads per group. We plan to recruit an additional 22 per group or 100 dyads for a total of 200 allowing for 22% attrition by 4 months [28].

### Randomization

Randomization will occur after the baseline assessment for each dyad (see Fig. 1). The randomization will be generated by a statistician external to the recruitment and the assessment process (LH). For concealment, group allocation will involve the use of opaque envelopes and will be conducted by a project manager who is not involved in any of the intervention or follow-up assessment processes.

### Intervention

The Healthy Patterns group will involve 4 in-home visits in the mid-morning and up to 4 brief telephone education sessions in the evening provided over 4 weeks. The Healthy Patterns intervention provides activities delivered at specific times in the daily cycle; it is theory-based, its components have been tested in pilot work; and it is portable and replicable (e.g., protocols are manualized). The in-home sessions are spaced weekly so that the participants can have the opportunity to practice the activity with the interventionist and then on their own. During each session the interventionist will reinforce activity use, review problem solving approaches and provide education. In Session 1 (home visit), the interventionist will explain the nature of the visit and assess the functional status and interests of the PLWD. The interventionist will then provide a suggested morning activity that will match the functional capabilities and interests of the PLWD i.e., based on repetitive motion (sorting objects) and integrating multi-sensory reminiscence stimulation (photos of interest). Activities selected are simplified (1 to 2 steps), to minimize errors. In Session 2 (home visit) the interventionist will review the implementation of morning activities and provide training materials for the afternoon physical activity. The afternoon physical activity will be based on the person’s level of physical functioning obtained at baseline and adapted from the Otago Exercise program [29]- an evidence-based, tailored, home based, balance and strength training program for older adults. In session 3 (home visit) the evening relaxation protocol is reviewed and includes a brief 10-step reflexology intervention in which pressure is applied to each of the reflex points on the feet for 30 s at a time for a total of 10 min. The protocol sequence is based on best practice guidelines. Caregivers are provided a picture book and video demonstrating the procedure. In Sessions 4 (one home visit) the interventionist will review integration of morning, afternoon, and evening activities into daily schedule and will provide written instruction on environmental cues to promote activity and sleep.

The interventionist has been trained in the Healthy Patterns protocol, and fidelity checks will be conducted to ensure appropriate adherence to the protocol will be conducted at random intervals. We will use fidelity monitoring strategies described by the NIH Behavior Change Consortium [30]. A treatment manual was developed to provide clear description of the delivery components of the intervention. Written records of all home sessions and phone calls will be collected. The treatment manual and videos demonstrating the activities are also available on a tablet that is given to each dyad. The tablet is reviewed by the study team to assess when the dyads view each of the activities. In addition, the interventionist and the PI and her team, will review randomly selected de-identified cases weekly.

#### Attention control protocol

The attention control condition will contain no active elements beyond its nonspecific components, and has no theoretical basis to support an effect on SWD. The control condition will be delivered by trained RAs who will provide social attention and engagement similar in duration to that provided to the Healthy Patterns Intervention group. The attention-control group will also involve 4 in-home visits in mid-morning. Each session is prescriptive and designed to maximize attention; yet sessions will not involve any of the components of the timed planned activity. The attention control group will receive printed Alzheimer’s Association and NIH materials on home modification, health promotion/ talking to your doctor and advanced care planning that coincide with session content. In Session 1 (home visit), the attention control interventionist will explain the nature of the visit and provides information on home safety. In Sessions 2–3 (one home visit and one phone call) the interventionist will work together with caregivers to answer questions about home safety and review health promotion material. In Sessions 4–5 (one home visit and one phone call), the interventionist will provide and review information on advanced care planning. Session 6 is a wrap up and review provided in the home. Time with the attention-control RA will be documented on a tracking sheet for each session. Weekly supervisory sessions with attention-control RAs will involve case presentations to track content of the attention-control sessions. In our previous studies, we found that the attention-control intervention is well received and that participants fully participated in sessions.

#### Measurement occasions

The week before the intervention begins, baseline measurements take place (T1). The same measurements take place after the final intervention visit at week 4 (T2). A follow up telephone measurement will occur 3 months after the final intervention visit (T3) Outcome measures are the same for all participants (see Table 1). Primary outcome measures are described in more detail under the heading ‘Materials’.

**Table 1:** Study Measures and Testing Occasions

Domain (purpose)	Measure	Description	Respondent	Testing Occasion
PLWD cognitive status (descriptive, possible covariate)	Clinical Dementia Rating (CDR) [27]	Cognitive status	PLWD	Nurse visit
PLWD physical function (descriptive, possible covariate)	Barthel Index [31]	CG proxy report of PLWD dependence level	Caregiver	T1
PLWD Quality of life as rated by CG (primary outcome)	QOL-AD [32]	12 domains	Caregiver	T1, T2
Sleep Wake disorder	Actigraph [33]	WASO, TST, day/night sleep ratio, number of night awakenings	PLWD	T1, T2
Subjective sleep PLWD	PROMIS Sleep Related Impairment [34]	Symptoms of sleep impairment in PLWD	Caregiver	T1, T2
Subjective sleep of caregiver	Pittsburgh Sleep Quality Index (PSQI) [35]	Sleep quality rating	Caregiver	T1, T2
HPA Axis activity (possible mechanism)	Salivary cortisol [36] Salivary melatonin [36]	Diurnal stress across the day	PLWD	T1, T2
Demographics (descriptive, possible covariate)	Caregiver and PLWD Information (US Census + other sources)	Basic background characteristics	Caregiver	T1
Medications (descriptive, possible covariate)	PLWD medications	Brown bag review of prescription and non-prescription meds	Brown bag review	Nurse visit
PLWD physical health (descriptive, possible covariate)	Charlson Comorbidity Index [37]	CG Proxy report of health	Caregiver	T1
Neuropsychiatric Behaviors (secondary outcome)	Neuropsychiatric Inventory (NPI) [38]	For each NPI behavior, CG rates upset (0 = no upset to 4 = very upset); Vigilance items ask CG to estimate time spent in care (alpha = .89)	Caregiver	T1, T2
Caregiver strain (secondary outcome)	Zarit Burden Interview (12) [39]	Caregiver burden	Caregiver	T1, T2
CG Depressive symptoms (potential moderator)	CES-D short form [40]	10-items; sensitive to change; Cut off for depression ≥8	Caregiver	T1, T2
Evaluation of study/ Quality control (descriptive, quality control)	Program Evaluation (Adapted from investigator’s previous studies)	Caregiver satisfaction with and utility of intervention; quality assurance	Caregiver	Telephone survey following T3

Note: *PlwD* person living with dementia, *T1* baseline, *T2* 1-month follow-up, *T3* 2-month follow-up. We estimate interviews with to average 1 h

### Materials

#### Primary outcome measures

SWD: Sleep-wake measures in this study include both objective (actigraphy) and subjective measures (Table 1). PLWD will be provided instructions on how to wear actigraphs (Philips Respironics Actiwatch Spectrum PRO) on their non-dominant hand to assess sleep-wake patterns for 28 consecutive days and nights. Wrist actigraphy is a reliable and a sensitive accurate method for measuring sleep wake disturbances including total sleep time, sleep latency, and wakefulness after sleep onset when compared to polysomnography [41, 42]. Caregivers will also be asked to complete a sleep diary that captures daily sleep patterns in PLWD [33] as recommended by the American Academy of Sleep Medicine [43]. The wrist actigraphs will collect data in 60-s epochs via tri directional accelerometer using an established algorithm. To analyze the data, we will use Actiware software, and the data will be reviewed by NH and NG and trained research staff. The primary measure of interest will be number of minutes of wake after sleep onset (WASO). Other objective sleep wake parameters that will be subject to analysis include total sleep time (TST), day/night sleep ratio, and number of night awakenings. Caregivers will be asked to use the event marker on the wrist actigraph to indicate “lights out” and “lights on” time.

Quality of Life: PLWD and caregiver perspectives of QOL will be collected using the dementia-specific 13-item Quality of Life in Alzheimer’s Disease scale [32]. Items reflect multiple dimensions (physical, social, emotional, and functional well-being) with each rated as 1 = poor, 2 = fair, 3 = good, or 4 = excellent. A total score representing the sum of items ranging from 13 to 52 will be derived separately for PWD and caregivers.

HPA Axis activity: Neuroendocrine activity will be assessed using the biological signature of the HPA response via daily salivary cortisol and an evening parameter of neuroendocrine activity via salivary melatonin. Based on current knowledge regarding diurnal patterns of PWD, caregivers will be instructed to collect saliva at the awakening challenge and at 3 additional intervals (30 min after waking, mid day, and at bedtime 1 h after dim light exposure [36]. Caregivers will be provided all materials and marked plastic bags to freeze samples until pick up by the trained data collector using a protocol published by the PI [44]. For T1 collection, saliva materials and instructions will be provided during the Screen for Disordered Breathing visit. Caregivers will be asked to collect saliva for 2 consecutive days prior to the intervention. For the T2 collection, caregivers will be asked to collect 2 days of saliva following the intervention (Session 8). Data collectors will call caregivers to review saliva sample collection and questionnaire packet procedures to assess for accuracy in completion, will clarify procedures as needed, and will send reminder text messages if requested. Data collectors will transport saliva samples on ice back to the study team’s dedicated − 40 degree freezer until shipment to the Institute for Interdisciplinary Salivary Bioscience Research (IISBR).

We will screen for depression in older adults via proxy caregiver using a validated questionnaire [40]. The Clinical Dementia Rating Scale will be used to estimate the level of cognitive impairment at baseline and as a covariate in the analysis [27]. We will also collect baseline demographic information including age, sex, race/ethnicity, relationship status, income, nature of the dyadic relationship, and years of education (See Table 1).

### Analyses

First, we will conduct descriptive analyses and univariate comparisons of the experimental and control intervention group conditions. We will use Chi-square, Wilcoxon rank-sum tests, and independent groups t-tests as appropriate to identify any differences between experimental and control group participants at baseline. In addition to serving as a final data quality check, these analyses will be used to characterize our study population, assess the success of randomization in balancing the two groups and determine the impact of any dropouts on group balance. Our primary analysis will examine the effect of the timed intervention compared to the attention control condition based on “intention to treat” (ITT). Data from all dyads randomized to the experimental group will be part of analyses regardless of actual number of completed intervention sessions. We will test the normality assumption for the SWD outcome measures (WASO, TST, neuropsychiatric symptoms; QOL) by examining the distribution of residuals after standard parametric comparisons. If the residual distribution is markedly skewed, we will examine whether data transformations are sufficient for improving the fit of the data to the normality assumption.

#### Aim 1

To analyze the effects of the timed intervention on our primary outcomes difference scores (T2 -T1) will be calculated for each participant and these change scores will be analyzed as a function of the treatment group, with the baseline observation (T1) serving as a covariate. Other potential covariates measured at baseline (e.g., age, education) will also be evaluated as potential predictors of change in outcome, and included as additional covariates of the intervention effect as indicated.

#### Aim 2

To test the effect of the timed activity intervention on caregiver outcomes. For Aim 2, we follow the approach for Aim 1 by examining group differences in the baseline-adjusted change scores (T2-T1) for caregivers outcome measures.

#### Aim 3

To test the mediating effect of patient HPA axis activity on symptoms of SWD, we will test mediation by modeling the measures of baseline-adjusted changes in SWD (WASO, TST, neuropsychiatric symptoms) in nested analytic models that either do or do not included the proposed mediator (e.g. T2 -T1 change in saliva cortisol). Using nested regression models, estimates will be obtained of 1) the effect of the intervention on baseline-adjusted change in cortisol, 2) the effect of the change in cortisol on baseline-adjusted change in WASO (or other outcomes), 3) the effect of the intervention on baseline-adjusted WASO before adding change in cortisol to the model, and 4) the effect of the intervention on baseline-adjusted WASO after adding change in cortisol to the model. These path estimates are often referred to by the letters a, b, c, and c in the mediation literature [45]. The product of the a and b estimates (a*b) represents the mediated effect, and this will be tested for statistical significance using the standard Sobel method or with bootstrapping techniques. The proportion of the total effect (c) that is explained by mediation (a*b) will also be calculated and tested for significance. Mediation models for data from two-wave randomized trials can provide substantially different results depending on whether change scores are used and whether baseline covariate adjustments are implemented. Our approach will provide sensitive analyses that determine whether the interventions impact on 1-month change in SWD symptoms is partly due to its effect on 1-month change in cortisol.

## Discussion

We hypothesize that scheduling activities based on shifting diurnal patterns across the day such as using cognitively stimulating activities in the mid-morning hours, physically stimulating activities in the afternoon hours and relaxing activities in the evening hours may help to reduce sleep-wake disruption in home dwelling PLWD. The success of the Healthy Patterns intervention will be measured by its impact on outcomes including measures of QOL and SWDs symptoms, including waking after sleep onset, day/night sleep ratio, and total sleep time. Caregiver outcomes of interest will be burden, subjective sleep quality, confidence in using activities and QOL. We will also examine the possible mechanism of action of timed activity interventions on SWD symptoms through diurnal neuroendocrine activity measured via salivary cortisol and melatonin. Results from the proposed study will provide fundamental new knowledge regarding the effects of timing activity participation based on diurnal needs and the mechanisms underlying timed interventions which can lead to a structured, replicable treatment protocol for use with this growing population of PLWD.

Since the study award and the University of Pennsylvania Institutional Review Board approval, we have enrolled and randomized 1 hundred dyads. We anticipate to complete recruitment in 2021 and the study results will be available by January 2022. Challenges thus far in the study have been during the recruitment stage. We have found that several potential participants are excluded because of a lack of a caregiver designee, as PLWD may be listed in our available data bases even if they do have a person who is designated as ‘caregiver’. As a team, we began to reframe our idea of who is being recruited, and started targeting caregivers more than PLWD. To decrease attrition, we will use several strategies such as beginning intervention within 2 weeks of recruitment, sending thank you, birthday and holiday cards as well as maintaining strong relationships with community partnerships. A number of participant payments will be offered to incentivize the participants to sustain participation. To minimize any missing data from the actigraphs and increase compliance we will call caregivers reminding them to complete sleep diaries, and to guide PLWD to wear the actigraphs.

Lastly, a diversity supplement has been added, titled Meaningful Activity and Quality of Life in older Latinos with Dementia (PI: AP). This purpose of this study is to culturally adapt the major elements of the Healthy Patterns Sleep Study, with two phases in place to do so: Phase I aims to use data from focus groups among older Latinos with dementia and their caregivers to adapt the intervention, and Phase II aims to examine the feasibility and proof of protocol with a pilot sample of this specific demographic.

Sleep-wake disorders are debilitating and contribute to poor quality of life for more than 5 million older PLWD and their caregivers [46–48]. Despite the prevalence of sleep disruption in PLWD, there are very few evidence-based, widely available non-pharmacologic interventions to improve sleep quality in this vulnerable population. The results from this study can provide fundamental new knowledge regarding the effects of timing activity participation and the mechanisms underlying timed interventions which can lead to a structured, replicable treatment protocol to address sleep wake disturbances.

## Data Availability

The datasets generated and/or analysed during the current study will be available from the corresponding author on reasonable request.

## Abbreviations

CONSORT: Consolidation Standards of Reporting Trials
HPA: Hypothalamic- pituitary-adrenal
NPI: Neuropsychiatric Inventory
PLWD: Person living with dementia
QOL: Quality of life
RA: Research Assistant
RCT: Randomized control trial
SWD: Sleep wake disorder
TST: Total Sleep Time
WASO: Wakefulness after sleep onset
